# Foreword to the special issue on new developments in structural stability

**DOI:** 10.1098/rsta.2022.0038

**Published:** 2023-04-03

**Authors:** S. Gerasimidis, J. W. Hutchinson, J. Sieber, J. M. T. Thompson

**Affiliations:** ^1^ Department of Civil and Environmental Engineering, University of Massachusetts Amherst, Amherst, MA 01003, USA; ^2^ School of Engineering and Applied Sciences, Harvard University, Cambridge, MA 02138, USA; ^3^ College of Engineering, Mathematics and Physical Sciences, University of Exeter, Exeter EX4 4QF, UK; ^4^ Department of Applied Mathematics & Theoretical Physics, Centre for Mathematical Sciences, Wilberforce Road, Cambridge CB3 0WA, UK

**Keywords:** shells, stability, buckling

This issue of the *Philosophical Transactions of the Royal Society* contains 12 papers detailing and employing methods under development for thinking about, analysing, and testing light-weight structures prone to buckling. Much of the motivation underlying the recent developments has emerged from efforts to improve methods for analysing buckling behaviour of thin shell structures carrying compressive loads. While there was extensive research into shell buckling which reached its peak about half a century ago, long-standing issues remain unresolved largely concerned with post-buckling behaviour and the sensitivity of buckling to structural imperfections. In recent decades there has been an expansion of interest in post-buckling behaviour driven by a wide array of new applications in problems investigating the functional behaviour of biological structures and soft materials in buckled states. Most of the earlier developments in buckling centred on the maximum loads beam, plate and shell structures could safely support. From this vantage point, buckling is something to be avoided. Many buckling studies now address nonlinear post-buckling behaviour with the aim of obtaining desirable structural or material responses for applications well beyond the scope of traditional structural theory. To highlight these two distinct attitudes toward buckling, Reis [1] has termed them ‘Buckliphobia’ and ‘Buckliphilia’.

For well over a century, it has been widely appreciated that, depending on the loading and support conditions, thin shells may buckle in a catastrophic manner with total collapse or at least with a dramatic reduction in load carrying capacity. Associated with this abrupt loss of load carrying capacity is an exceptionally strong imperfection-sensitivity whereby relatively small departures from the assumed perfect shell geometry, support conditions or applied loads can significantly reduce the load carrying capability below that predicted for the perfect structure. Two canonical examples are cylindrical shells under axial compression and spherical shells under external pressure. A large body of experimental data gathered over the past century has revealed that the buckling loads of these two structure/load combinations have enormous scatter with buckling loads as low as 20% of the prediction for the perfect shell. Much of the past research has focused on shells that are sufficiently thin that they remain elastic, at least prior to attainment of the maximum load. For most structural metal alloy, polymer or composite shells, the strains at buckling cannot exceed a fraction of a percent if buckling is to be elastic, which, in turn, requires the shell radius to thickness ratio to be not less than about *R*/*t* = 100, depending on the yield strength of the material. Many aerospace and some civil engineering shell structures are designed to remain elastic and have radius-to-thickness ratios as large as 1000 or more.

The buckling strength advantage of thin shells over beam and plate structures can be enormous, as illustrated in figure 1 by ratio of the elastic axial buckling loads of a perfect thin-walled square plate column to that of a circular cylindrical shell made of the same material with the same mass and length *L*. The thinner the walls of the structures, the greater the relative buckling strength of the shell. The buckling mode of the square plate tube is a regular pattern of square buckles correlated around the circumference and along the length with a half-wavelength equal to the width *w*. The buckling mode of the cylindrical shell is typically a diamond shaped pattern covering the shell which has a shorter wavelength proportional to $\sqrt{Rt}$. This huge strength differential comes with a penalty. As depicted in the figure, the plate tube has a stable post-buckling behaviour such that the structure can support loads above the buckling load of the perfect structure. Some applications have exploited this additional capacity. By contrast, the perfect shell column abruptly loses its load carrying capacity at the onset of buckling and the load it can support in the post-buckling state is a fraction, often a small fraction, of the buckling load. Coupled to this catastrophic post-buckling behaviour of the perfect shell is an extreme sensitivity to imperfections, elucidated analytically by Koiter [2], wherein the maximum attainable support is severely reduced below the buckling load of the perfect shell. Although it buckles at a much lower load, the square plate tube is relatively insensitive to imperfections and can even support additional load.

**Figure 1.**
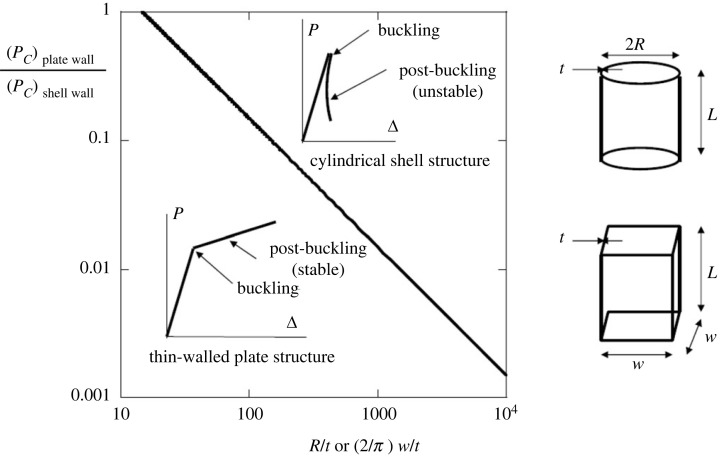
Ratio of the elastic axial buckling loads of short tube-columns of length *L* for a square thin-walled flat plate structure to that of a thin-walled cylindrical shell, each having the same wall thickness *t*. The columns are made from the same linear isotropic elastic material with a Poisson ratio, *ν* = 1/3 . The ratio is plotted assuming equal mass in the two columns which requires *R* = (2/*π*)*w*. The ratio applies, at least to a good approximation, if the length is more than about 3 or 4 times the width *w* and radius *R*, but not for very large *L* when the columns buckle in the Euler mode. The ratio is computed assuming each structure is perfect with simply supported conditions at the ends, but the ratio remains approximately applicable for clamped end conditions if *L* is greater than 3 or 4 times the width and radius. The load, *P*, versus end-shortening, Δ, behaviour of each of the two perfect structures is indicated by a sketch.

The local loss of shell curvature as the shell buckles undercuts the enormous strength advantage of the cylindrical shell in figure 1 and accounts for the abrupt drop in load carrying capacity shown in the sketch. Nearly all the early work analysing the post-buckling behaviour of shell structures dealt with modes in the shape of the classical buckling modes (the eigenmode or modes) which usually extend over the entire shell, or at least over a large portion of the shell. Similarly, previous imperfection-sensitivity studies almost always considered geometric imperfections in the shape of the regular patterns set by the classical buckling modes. In large part, this focus was due to a reliance on analytical methods and the limitations at the time of available numerical computational methods and the large size of the computations. It has only been in relatively recent years that research has focused on localized post-buckling behaviour and, to a lesser extent, on localized imperfections, even though localized buckling behaviour had been commonly observed in the earliest experiments and it was widely appreciated that imperfections in the shape of the eigenmodes extending in a highly correlated manner over large regions of the shell were unrealistic. Several of the authors contributing papers to this special issue have been instrumental in generating this shift of focus. As will be cited in papers in this issue, one of the important discoveries for the elastic buckling of cylindrical shells under axial compression is the existence of stable localized post-buckling modes for the perfect shell at loads in the range of 40 to 60% of the classical bifurcation load. In other words, a buckled perfect cylindrical shell can support loads well above the conservative estimate of roughly 20% of classical load that is predicted by an analysis formulated for non-localized modes and observed for some shells in the experimental data set.

In addition to the scientific discoveries that have revealed the broader range of buckling behaviours, other motivations are driving current research on the stability of imperfection-sensitive structures. The original design codes on which much of the design of lightweight shell structures still rests emerged in the 1960s. Efforts are underway in China, Europe and the USA to update these codes. The design load of about 20% of the classical buckling load for thin cylindrical shells under axial compression and spherical shells under external pressure is widely regarded as unrealistically low for well-constructed shells, and it is one of the targets for revision. It is important to appreciate that the 20% factor, known to the buckling community as the knockdown factor, is not a safety factor. It is regarded as the estimate of the buckling load, and any safety factor must be added on top of the knockdown factor. Since the early design codes were put forth, thanks to advances in computers and computational methods, the commercial structural analysis computer codes are now capable of generating accurate engineering predictions for the buckling loads of imperfection-sensitive structures if accurate representations of the imperfections are input to the codes. In addition, manufacturing methods for shell structures have advanced significantly with the potential to produce shells meeting more stringent tolerances. Techniques for measuring imperfections have also improved. Thus, the recent advances in shell buckling analysis noted above play nicely into the overall ambitions of the structural stability community, and this special issue presents original research furthering this aim.

The papers in this issue deal with various aspects of nonlinear buckling behaviour, but the two main concepts that underlie much of the attention are post-buckling behaviour and the associated energy landscapes and barriers, and the technique of probing a loaded structure prone to unstable buckling to gain insight into its nonlinear behaviour and to estimate the load at which it will buckle. Except for two papers in this issue, all the papers deal primarily with elastic buckling. Almost two-thirds of the papers have a substantial experimental component.

Probing to explore the propensity to buckle is at the heart of the first five papers in this volume and these papers cite the earliest efforts to apply this method. Although probing loaded shell structures goes back many years, the idea of using probing in a systematic manner as a tool for exploring stability was promulgated only recently by Thompson [3] and Thompson *et al*. [4]. In this issue, Lachman *et al*. [5] conduct experimental probing of a shallow arch to illustrate the salient features of the technique for a structure that has a rich buckling behaviour but is not as complex as most shell structures. Their paper also introduces terminology relevant to probing the energy landscape of the nonlinear behaviour. Discovering structural response by probing can be an effective technique whether it is conducted experimentally or computationally, as will be evident in the first six papers. The paper by Royer *et al*. [6] on the ultra-lightweight structures used for supporting solar cell arrays in space employs both experimental and computational probing to reveal the highly complex buckling behaviour of these structures. The following quote from the abstract of this paper succinctly encapsulates the objectives of the technique: ‘These results can be used to formulate efficient buckling criteria and pave the way to operating these structures close to their buckling limits, and even in their post-buckling regime, therefore significantly reducing their mass.’

The papers by Cuccia *et al.* [7] and Groh *et al*. [8] deal with application of the probing technique to cylindrical shells under axial compression. The first paper examines the elastic buckling of thin shell soda cans that have been deliberately dented. A background imperfection pattern inherent to each can is such that even without the deliberate imperfections, the shell buckles well below the prediction for a perfect can. This is one of the first papers to explore the importance of probing location for a structure with complex buckling behavior. It shows that depending on the severity of the deliberately introduced imperfection it may or may not initiate buckling, and the probe survey of the shell must be conducted with sufficient resolution to be able to ascertain the most likely site for initiation and the associated buckling load. In one of the most advanced applications of computational probing yet to be published, Groh *et alo*. [8] perform a thorough exploration of the energy landscape of perfect cylindrical shells under axial compression identifying loads associated with stable localized buckles. These authors have used their results for the cascade of the stable post-buckling modes to suggest trends for the buckling knockdown factor as a function of the Batdorf parameter. While their results are derived for perfect cylindrical shells, the authors emphasize that aspects of the trends appear to be in accord with the large experimental dataset for buckling of imperfect shells, and they argue that the probing results provide a ‘more nuanced’ understanding of post-buckling behaviour. We return to the issue of inferring buckling load capacity of imperfect structures from the behaviour of the perfect structure at the end of this Foreword.

Prestressed stayed columns can display unstable post-buckling behaviour and strong imperfection-sensitivity, especially when they have been optimized to maximize the buckling load of the perfect structure. The paper by Shen *et al*. [9] outlines a comprehensive structural health monitoring approach to prestressed stayed columns based on probing. This paper provides another excellent illustration of the power of the probing method for estimating the propensity for buckling and a compelling case for the practical utility of the method. In addition, the authors discuss implementing machine learning to extract structural health indices from the probing data conducted over the lifetime of the structure.

Derveni *et al*. [10] carry out a combined experimental and computational study of the buckling interaction between localized bump imperfections (outward nonuniformities from the perfect geometry, rather than inward dimples) distributed randomly over the surface of thin hemispherical shells clamped at their equator and subject to external pressure. The study explores aspects of the stochastic nature of the buckling process and gives insight into when interaction between the bump imperfections is significant and when it is not. This is a follow-on of earlier studies led by the senior author which have provided the most convincing evidence so far that accurate predictions of the buckling load can be computed if imperfections of actual shells are carefully measured and input into an established nonlinear shell finite-element code.

Localization of buckling deformations immediately after the onset of buckling often accompanies the falling load-displacement response of shells and other imperfection-sensitive structures. Kumar *et al*. [11] analyze the bending behaviour of open, curved strip shells such as those used in tape measures and Venetian blinds. Under bending, these long lightweight structural elements dramatically lose their bending strength as they buckle because the buckling deformation lowers the shell curvature over one or more local regions along the strip. The authors make use of dimensional reduction methods to reduce the nonlinear partial differential equation governing the buckling to a much simpler ordinary differential equation that accurately captures the localization and the progressive loss in bending stiffness. The authors and their co-workers have applied dimensional reduction to other nonlinear systems with great success, and the contribution in this paper should prove useful in modelling systems comprising the shell strip beams such as those employed as the structural elements in the space frames designed for the solar arrays of Royer *et al*. [6].

The last five papers in the special issue are illustrative of new applications involving buckling which bring to bear the analysis and experimental tools highlighted in this issue to design and explore the nonlinear buckling response of new structures. The paper by Abassi *et al*. [12] provides design, analysis, and experimental testing of bistable compressed elastomeric columns driven from one stable state to the other by an external magnetic field that acts on magnetized particles that are embedded in the column. This paper serves as an exemplar of modern developments in stability theory. Solutions based on finite-element analysis with elements distributed along the length and through the thickness are obtained but, in addition, a simpler and more insightful reduced one-dimensional nonlinear beam formulation is also derived and demonstrated to accurately reproduce the FEM predictions. All aspects of the behaviours explored in this paper are validated by experiments. The paper includes a section on the use of probing to experimentally estimate when the columns will snap from one state to the other, demonstrating that this technique works well for the bistable structural elements.

The paper by Chabouh *et al*. [13] on the buckling of micrometer size bubbles encased by a thin lipidic shell in a fluid is an excellent example of the extraordinary variety of applications of buckling theory that have appeared in recent years at many size scales. Bubbles encased by lipidic shells have important biomedical applications, for example by modifying the ultrasonic response of blood in a systematic manner useful as a diagnostic tool. The paper by Chabouh *et al*. [13] is a multi-physics study of the buckling behaviour of these bubble/shell entities, combining analysis and experiments, motivated primarily to establish the validity of the models used to characterize them and to measure the properties of the lipidic layer and other components of which they are comprised. The behaviour of these shell bubbles, like many shell structures, is rich in its mechanics. The shell bubbles display viscoelastic behaviour and the authors have examined buckling under oscillatory excitation as well as quasi-static conditions.

Cooley *et al*. [14] have used three-dimensional printing to manufacture laboratory-scale cylindrical shells with well-defined geometric imperfection patterns. The main thrust of the paper is to convey the advantages of three-dimensional printing for experimental investigations of shell buckling given the ability of the experimentalist to manufacture shells with a wide range of relevant parameters and different types of precisely defined imperfections. The shells manufactured and tested in this study fall with the range of practical interest with length to radius ratios in the range from about 2 to 5, radius to thickness ratios between 100 and 150. A variety of geometric imperfection patterns are manufactured, including cylinders with a reduced waist, with sinusoidal eigenmodal shapes and local dimple imperfections. A limited study of probing of several axially loaded shells is also performed to demonstrate the potential of the technique for the nondestructive determination of the buckling load.

Lincoln *et al*. [15] conduct a study of fibre reinforced composite cylindrical shells under axial compression with an eye to choosing a fibre/tow layup method so as to reduce the inherent imperfection-sensitivity of the shells. This work is heavily informed by the recent advance in understanding related to the lowest energy paths of an axially loaded cylindrical shell from the pre-buckling state to stable buckled states. It also draws parallels to the role that stiffeners play increasing buckling strength while at the same time reducing imperfection sensitivity. The idea of taking advantage of the rapid tow shearing process to achieve these goals is shown to be promising with increased buckling strength compared to comparable composite shells of more conventional laminate design. This paper joins a small set of recent papers devoted to reducing imperfection-sensitivity by creative material and/or geometrical design rather than by directly lowering imperfection amplitudes in the manufacturing process.

The final paper in the issue by Chen *et al*. [16] explores the role of viscoelasticity in snap buckling of shells capable of supporting bistable states. Important time-dependent phenomena are identified and analysed in the paper such as delayed snapback and its dependence on the hold time prior to release of the load used to ‘set’ the buckle in the snapped state. Thus, this paper expands the scope of the papers by Lachmann *et al*. [5] and Abassi *et al*. [12] on elastic snap buckling of arched columns into the realm of shells with time-dependent material behaviour. The authors consider pressure and volume-change loadings of axisymmetric ellipsoidal shells with edge support conditions that give rise to snapping behaviour, and they focus their attention on the range of geometries for which the nonlinear buckling behaviour is axisymmetric. The paper includes a section on the use of probing conducted rapidly compared to the relaxation time of the material to explore the time-evolution of the stability prior to snap back.

As is evident from this Foreword, post-buckling behaviour and imperfection-sensitivity are two of the ongoing central concerns in structural stability and in this special issue. Efforts to draw conclusions concerning the buckling of imperfect versions of a structure from the post-buckling behaviour of the perfect structure go back to the earliest days of nonlinear shell buckling analysis, and they persist. Much of the recent work on shell buckling has continued to focus on the behaviour of perfect shells with qualitative arguments made as to the relevance to imperfection-sensitivity and buckling knockdowns. Of major importance in Koiter's [2] contribution to elastic stability was the quantitative coupling of buckling load reductions due to imperfections to the buckling and post-buckling behaviour of the perfect shell. In effect, Koiter showed how the post-buckling behaviour of the perfect structure serves as the analytical backbone for computing the load carrying capacity of imperfect versions. This special issue advances our understanding of the connection between the behaviour of a perfect structure and its imperfect realizations for stable localized modes and imperfections. Nevertheless, it is imperfections which give rise to buckling load reductions, and quantitative results concerning this connection for localized modes and imperfections remain limited.

## Data Availability

This article has no additional data.
